# Retraction and replacement of: Telomere-to-telomere genome and resequencing of 254 individuals reveal evolution, genomic footprints in Asian icefish, *Protosalanx chinensis*

**DOI:** 10.1093/gigascience/giaf066

**Published:** 2025-07-17

This is a retraction and replacement of Yanfeng Zhou, Chenhe Wang, Binhu Wang, Dongpo Xu, Xizhao Zhang, You Ge, Shulun Jiang, Fujiang Tang, Chunhai Chen, Xuemei Li, Jianbo Jian, Yang You, Telomere-to-telomere genome and resequencing of 254 individuals reveal evolution, genomic footprints in Asian icefish, *Protosalanx chinensis, GigaScience*, Volume 14, 2025, giae115, https://doi.org/10.1093/gigascience/giae115.

On 05 December 2024, *GigaScience* published an article entitled ‘Telomere-to-telomere genome and resequencing of 254 individuals reveal evolution, genomic footprints in Asian icefish, *Protosalanx chinensis* by Yanfeng Zhou, Chenhe Wang, Binhu Wang, Dongpo Xu, Xizhao Zhang, You Ge, Shulun Jiang, Fujiang Tang, Chunhai Chen, Xuemei Li, Jianbo Jian, Yang You.

Soon thereafter, the Editors were contacted by a reader who raised a concern that the (Chongming Island, CMD) samples were quite different from all other populations. And when the mitochondrial genome of the samples from CMD were extracted, it was evident that all the individuals were actually from *Salanx ariakensis*. The Editors obtained advice on this from a fish expert, who agreed with the reader that these individuals from Chongming Island (CMD) are *Salanx ariakensis*, so all the conclusions about these CMD samples from resequencing were incorrect.

The samples were checked again based on external morphological features. However, the samples were preserved in alcohol, which may affect the species identification. Thus, the authors have accepted the feedback provided by the reader and requested retraction and replacement of their article. The authors have agreed to retract the original version of the article and replace it with a new version with the group 0 samples (including CMD 18 individuals and 5 THL individuals) deleted and Figure 4 updated. The information related to the samples has been updated with the related Excel files. The CMD samples have been removed, resulting in a reduction of sample sites to seven locations. The old version of Supplementary Figure S5 and the geographic distribution of 7 sampling sites have been updated. The old versions of Supplementary Fig. S6, Fig. S7, Fig. S8, Fig. S10, Fig. S11 which were related to CMD have been removed. In the original version of the article, most of the results did not include CMD with the five samples from THL, and the analysis was conducted independently. Therefore, in this updated version, these results, which now encompass 231 samples, have been retained. The information of 23 samples (group 0) in Supplementary Table S16, Table S17, Table S18, Table S19, Table S20 have also been removed and the related tables have been updated.

The new version, “Telomere-to-telomere genome and resequencing of 231 individuals reveal evolution, genomic footprints in Asian icefish, *Protosalanx chinensis*” was judged to fully support the conclusions drawn. The new version was reviewed and accepted by the editors and has been republished at https://doi.org/10.1093/gigascience/giaf067

